# Deep Learning–Based Automated Clinical Gait Assessment From Kinematic Data in People With Stroke: Development and Validation Study

**DOI:** 10.2196/94031

**Published:** 2026-08-19

**Authors:** Zhexuan Gu, Jiaqi Li, Wenbin Zhou, Kenneth N K Fong, Lin Wang, Eng Keong Lua, Patrick W H Kwong, Yancheng Yuan

**Affiliations:** 1 Department of Data Science & Artificial Intelligence, Hong Kong Polytechnic University, Hong Kong, China (Hong Kong); 2 Department of Rehabilitation Sciences, The Hong Kong Polytechnic University, 11 Yuk Choi Road, Hung Hom, KLN, Hong Kong, China (Hong Kong), 852 3400 3958; 3 Shenzhen Institutes of Advanced Technology, Chinese Academy of Sciences, Shenzhen, China; 4 Department of Electrical and Electronic Engineering, University of Hong Kong, Hong Kong, China (Hong Kong); 5 Computer Laboratory, University of Cambridge, Cambridge, England, United Kingdom; 6 Department of Applied Mathematics, The Hong Kong Polytechnic University, Hong Kong, China (Hong Kong)

**Keywords:** deep learning, gait, graph convolution network, stroke, Wisconsin Gait Scale

## Abstract

**Background:**

Gait impairment is a prevalent sequela of stroke. Although observational gait analysis remains a standard clinical practice for assessing neuromotor impairments in people with stroke, it is prone to subjective bias. Consequently, objective assessment is required to inform effective rehabilitation protocols.

**Objective:**

This study aimed to validate a deep learning framework for automating clinical gait assessment using kinematic data.

**Methods:**

This study was conducted in a university-affiliated gait analysis laboratory. Kinematic data were collected from 51 individuals with hemiparetic stroke and 18 healthy controls using wearable inertial measurement units, and video recordings were obtained for the Wisconsin Gait Scale (WGS) scoring. WGS scores, rated by 2 experienced physiotherapists, were used to establish the expert reference standard. A statistical graph convolutional network (STAT-GCN), incorporating a STAT-Attention Head module, was developed to predict WGS items 2 to 14 from kinematic data. Model evaluation was performed using a stratified, participant-level 5-fold cross-validation protocol, with all gait cycles from the same participant kept within the same fold. Model performance was assessed at the participant level using exact accuracy, mean absolute error, and weighted κ metrics. A descriptive comparison involving 4 final-year physiotherapy students was also conducted.

**Results:**

Among participants with stroke, STAT-GCN achieved an overall exact accuracy of 75.42% for WGS items 2 to 14, with a mean absolute error of 0.290 score levels. Across the participant-level 5-fold cross-validation test sets, STAT-GCN achieved a mean exact accuracy of 75.64% (SD 6.58%), whereas the 4 student evaluators achieved mean accuracies ranging from 56.19% (SD 7.03%) to 67.09% (SD 4.03%).

**Conclusions:**

The proposed STAT-GCN framework demonstrated reasonable preliminary performance for automated WGS item prediction in individuals with stroke. The results suggest that kinematic data–driven models may support more standardized gait assessment, but performance should be interpreted cautiously given the limited sample size, class imbalance across WGS score items, and absence of external validation. Further studies with larger and more clinically diverse stroke cohorts are needed before clinical implementation.

## Introduction

Stroke is a principal contributor to global mortality and long-term disability [1,2]. It constitutes a major public health challenge, imposing substantial societal and health care burdens worldwide. The complications of stroke are debilitating and commonly include muscle weakness, spasticity, and limb apraxia, which contribute to gait impairments [3,4]. These impairments present as reduced cadence and speed, resulting in an abnormal gait pattern. These impairments may also lead to difficulties in maintaining balance [5-7]. Consequently, gait impairments affect the level of community integration in patients with stroke [8].

Observational gait analysis remains a conventional clinical practice for assessing neuromotor impairments in people with stroke; however, this methodology is prone to subjective bias in clinical assessments [9]. Thus, for greater systematicity, standardized gait analysis tools such as the Wisconsin Gait Scale (WGS) have been adopted to evaluate videotaped gait patterns in individuals with stroke [10]. The WGS [11] is a widely adopted assessment tool for evaluating gait abnormalities. It comprises 14 items, of which 13 are related to gait performance and gait patterns and are rated through observation of gait. The WGS is clinically efficient, requiring minimal instrumentation, and has demonstrated high intrarater reliability (intraclass correlation coefficient [ICC]=0.83) in poststroke validation studies [12]. However, manual scoring by a rehabilitation specialist is not only time-consuming but also subject to bias in subjective clinical judgment [13]. Furthermore, rating many WGS items requires the observation of subtle changes in joint angles, such as the rotation angle of a specific joint. The visual identification of these subtle changes is highly challenging.

AI has been increasingly used to automate and reduce subjective bias in clinical assessment [14,15]. A multimodal graph fusion network was proposed in a previous study [16] to detect the freezing of gait, a disabling symptom of Parkinson disease. The graph fusion network comprised 4 modalities, each corresponding to a distinct data source. Graph fusion networks have also been used successfully to determine the severity of parkinsonism [17,18]. Similarly, a study [19] successfully distinguished Parkinson disease cases from controls using a novel model, namely a multiview graph convolutional network (GCN). Human motion analysis represents an evolving computational paradigm where skeleton-based kinematic time-series data serve as critical inputs for emerging action recognition algorithms [18,20-22]. GCN-based models have demonstrated state-of-the-art performance on established motion analysis benchmarks, achieving >93% accuracy on the NTU RGB+D dataset [23]. However, translational applications in clinical gait assessment remain limited [24], primarily due to the fundamental disparity between action classification and quantitative clinical evaluation. Evaluating an action in clinical settings requires a more nuanced level of analysis than simple recognition, as it involves complex assessments of quality, precision, and clinical relevance [25]. Standard deep learning architectures, such as convolutional neural networks and recurrent neural networks, often struggle with this level of granularity because they treat skeletal data as flat sequences or grids, ignoring the intrinsic biomechanical connectivity between joints. This loss of topological information makes it difficult to quantify the subtle deviations in interjoint coordination that characterize pathological gait.

To address this gap, we propose a novel statistical graph convolutional network (STAT-GCN) for the automation of WGS scoring. By using gait kinematic data collected from inertial measurement units (IMUs) [26], this model enables the representation of joint angular trajectories as spatiotemporal features on a graph structure. By preserving the topological structure of lower-limb joints, this approach is intended to improve the model’s ability to learn clinically relevant gait features for automated WGS item scoring.

## Methods

### Ethical Considerations

This study was conducted in accordance with the Declaration of Helsinki and was approved by the Institutional Review Board of The Hong Kong Polytechnic University (approval number HSEAR20210715003). All participants provided written informed consent. Participants’ privacy and confidentiality were maintained throughout the study. All collected data were deidentified and stored securely, and only authorized members of the research team had access to the data. Participants received reimbursement for transportation expenses associated with study participation.

### Participants

This study enrolled individuals with stroke according to the following criteria: (1) aged 18 to 80 years, (2) diagnosis of ischemic or hemorrhagic stroke confirmed by magnetic resonance imaging or computed tomography more than 6 months before assessment, (3) able to walk 10 m independently or with assistance, and (4) capable of providing informed consent. Individuals were excluded if they had any medical conditions that hindered their ability to participate in the assessment. In addition, 18 healthy individuals were recruited according to the following criteria: (1) aged 18 to 80 years, (2) no history of stroke or other diseases that may affect gait patterns, (3) able to walk independently, and (4) capable of providing informed consent. From March to August 2024, eligible participants were recruited through convenience sampling with assistance from local self-help groups.

Because no consensus has been established on sample size calculations in studies focused on developing deep learning models for various clinical applications [24], we referred to our previous study, which included 40 participants. In addition, an AI model using gait kinematic data achieved an accuracy of 84% in predicting the outcomes of clinical assessments of functional mobility conducted by physiotherapists [14]. Based on the sample sizes of these previous studies, for the development of a deep learning model, our trial aimed to recruit 51 participants. The study protocol was conducted in accordance with the STROBE (Strengthening the Reporting of Observational Studies in Epidemiology) guidelines (Checklist 1).

### Outcome Measures

#### Gait Assessment and Clinical Measures

In this study, gait kinematics were measured in participants with stroke. Gait analysis and kinematic assessment were conducted using the MTw Awinda system (Xsens Technologies B.V.). This system consists of an IMU containing a 3D gyroscope, 3D accelerometer, and 3D magnetometer. Data were sampled at a frequency of 60 Hz. According to the official guidelines, body motion was captured by attaching 17 wireless sensors to the head, shoulders, sternum, upper arms, forearms, hands, pelvis, upper legs, lower legs, and feet of the individual. The IMUs were secured using MTw Velcro Body Straps and tape. Data were transmitted between the wireless tracker and the Awinda Station using the patented Awinda radio protocol, which was specially developed to ensure accurate 3D motion tracking even with unstable data transmission [27], and were processed using Xsens MVN 2024 software for gait analysis and kinematic assessment.

This study also used 2 distinct outcome measures:

1. WGS served as the primary outcome measure and our modeling target.
2. The Motor Subscale of the Fugl-Meyer Assessment of the Lower Extremity (FMA-LE) was used as a supplementary assessment to provide a broader characterization of participants’ motor impairment.

#### WGS

The WGS is a gait assessment tool specifically designed for individuals with hemiplegia (Multimedia Appendix 1). The scale comprises 14 items and is designed to measure clinically relevant gait patterns during the stance phase, toe-off, swing phase, and heel strike of the gait cycle. Rodriguez et al [28] first introduced the WGS using videotaped gait recordings obtained in the frontal and sagittal planes. Each item is rated on an ordinal scale of either 1 to 3 or 1 to 4. The total score ranges from 14 to 45, with higher scores indicating greater gait impairment. As the primary outcome measure, WGS scores were assigned based on participants’ gait. The ground truth was established based on consensus WGS scores agreed upon by 2 Hong Kong–registered physiotherapists, each with more than 10 years of clinical experience. Additionally, 4 final-year physiotherapy students provided WGS ratings to represent the perspectives of inexperienced raters.

#### FMA-LE

As a supplementary measure to characterize participants, the FMA-LE was used. Although the full FMA-LE evaluates motor function, sensation, range of motion, and pain [29], the motor subscale specifically is widely used to assess poststroke lower extremity motor impairment. This subscale evaluates reflex activity, movement in and out of synergy, as well as coordination and speed. It comprises 17 items, each rated on a 3-point ordinal scale (0‐2), summing to a maximum total score of 34 (indicating normal function) [30]. It demonstrates excellent intrarater reliability (ICC=0.99) and interrater reliability (ICC=0.91) [31].

### Study Procedures

Prior to kinematic analysis using the Xsens MVN 2024 software, participants with stroke underwent a standardized assessment using the motor subscale of the FMA-LE [32]. Subsequently, following the Xsens user manual [33], 17 sensors were attached to the corresponding body segments of each participant for motion capture. Participants were instructed to walk back and forth in a straight line at a self-selected, comfortable speed over a flat 10 m surface. Three trials were recorded, ensuring that participants completed at least 5 full gait cycles in each direction. All walking trials were videotaped in both the sagittal and frontal planes. Finally, each trial was reviewed by 2 experienced physiotherapists to generate a consensus WGS score. The gait kinematic data exported from the software were then used to develop a deep learning model, namely, the STAT-GCN model, to predict WGS scores for participants with stroke. Item 1 of the WGS (ie, use of a walking aid) was excluded from the analysis because this item is easier to rate through direct observation.

### Model Training and Testing

#### Cross-Validation and Data Partitioning

To ensure a rigorous evaluation of the proposed model, we implemented a stratified, participant-level 5-fold cross-validation protocol across the entire cohort (51 individuals with stroke and 18 healthy controls). The dataset partitioning was executed strictly at the participant level; that is, all gait cycles originating from a single participant were assigned exclusively to either the training set *D*_train_ or the testing set *D*_test_. This participant-isolated strategy guarantees that the model was tested solely on unseen individuals, thereby eliminating the risk of intraparticipant data leakage.

The cross-validation folds were stratified by clinical groups to maintain an approximately balanced proportion of patients and controls across the folds. During the inference phase, a majority-voting mechanism was applied to aggregate cycle-level predictions into a definitive participant-level clinical score. The most frequent prediction across all available gait cycles for a given participant was designated as the final predicted score for each WGS item.

Data augmentation was applied only to the training gait cycles within each cross-validation fold [34]. Three time-series augmentation strategies were used to simulate natural gait variability and sensor measurement noise: jittering, scaling, and time warping. Jittering added small Gaussian noise to the original gait trajectories, scaling adjusted feature amplitudes to represent variations in stride intensity, and time warping introduced smooth temporal distortions to simulate cadence fluctuations within a gait cycle. Through these augmentation procedures, the number of training gait cycles increased from 979 to 10,152. No augmented samples derived from test participants were included in the training set, and test set performance was evaluated using nonaugmented gait cycles from unseen participants.

To reduce the risk of overfitting, the STAT-GCN was designed as a compact model. It consists of 3 spatiotemporal graph convolutional blocks followed by a statistical head, with channel widths of 24, 48, and 48 across the 3 blocks. The STAT-Attention module was implemented as a lightweight feature-scaling mechanism rather than a dense pairwise self-attention layer. The same model configuration and hyperparameter settings were applied across all cross-validation folds. The model was trained with a batch size of 16 for 40 epochs. The initial learning rate was set to 1×10⁻³, with a step decay factor of 0.8, and the weight decay was set to 0.1 for L2 regularization. No fold-specific hyperparameter tuning or test set–based model selection was performed.

#### Backbone Network

For our model, a batch of skeleton data, D∈R^(B×C×T×J)^, is used as input, where B is the batch size, C represents the number of raw features, T represents the number of frames in 1 gait cycle, and J is the number of joints. The backbone network is adapted from previous studies [18,35], with an additional statistics module.

The role of a spatio-temporal graph convolutional network (ST-GCN) block is 3-fold. It embeds low-level features into a high-dimensional hidden space, facilitates the exchange of information between joints, and aggregates features along the temporal dimension for each joint. By stacking multiple ST-GCN blocks, the model can gradually expand its receptive field and learn meaningful features.

#### STAT-GCN

**Figure 1.**
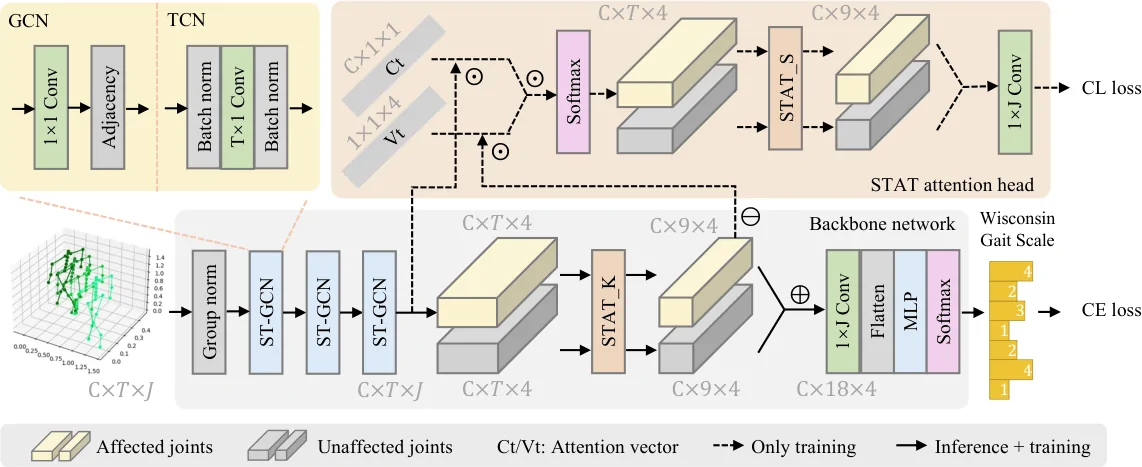
Proposed STAT-GCN architecture. CE: cross-entropy loss; CL: contrastive loss; Ct: channel attention vector; MLP: multilayer perceptron; ST-GCN: spatio-temporal graph convolutional network; STAT-GCN: statistical graph convolutional network; TCN: temporal convolutional network; Vt: vertex attention vector (graph vertex=joint).

To address the inadequate performance of prior data-driven approaches due to limited clinical data, we propose STAT-GCN (Figure 1). This model processes input skeleton data derived from motion capture to predict WGS scores. The inference architecture consists of a backbone network built from multiple ST-GCN blocks followed by a statistics module. To enhance feature discrimination, we introduce a STAT-Attention Head (SA Head) exclusively during training. This module uses contrastive loss and attention mechanisms to explicitly extract critical statistical features and highlight significant joints. Furthermore, by leveraging clinical labels regarding the affected side in participants with stroke, we segregate joint features into “affected” and “unaffected” categories to minimize misinterpretation. Consequently, STAT-GCN generates highly representative features tailored to each patient’s specific mobility characteristics.

**Figure 2.**
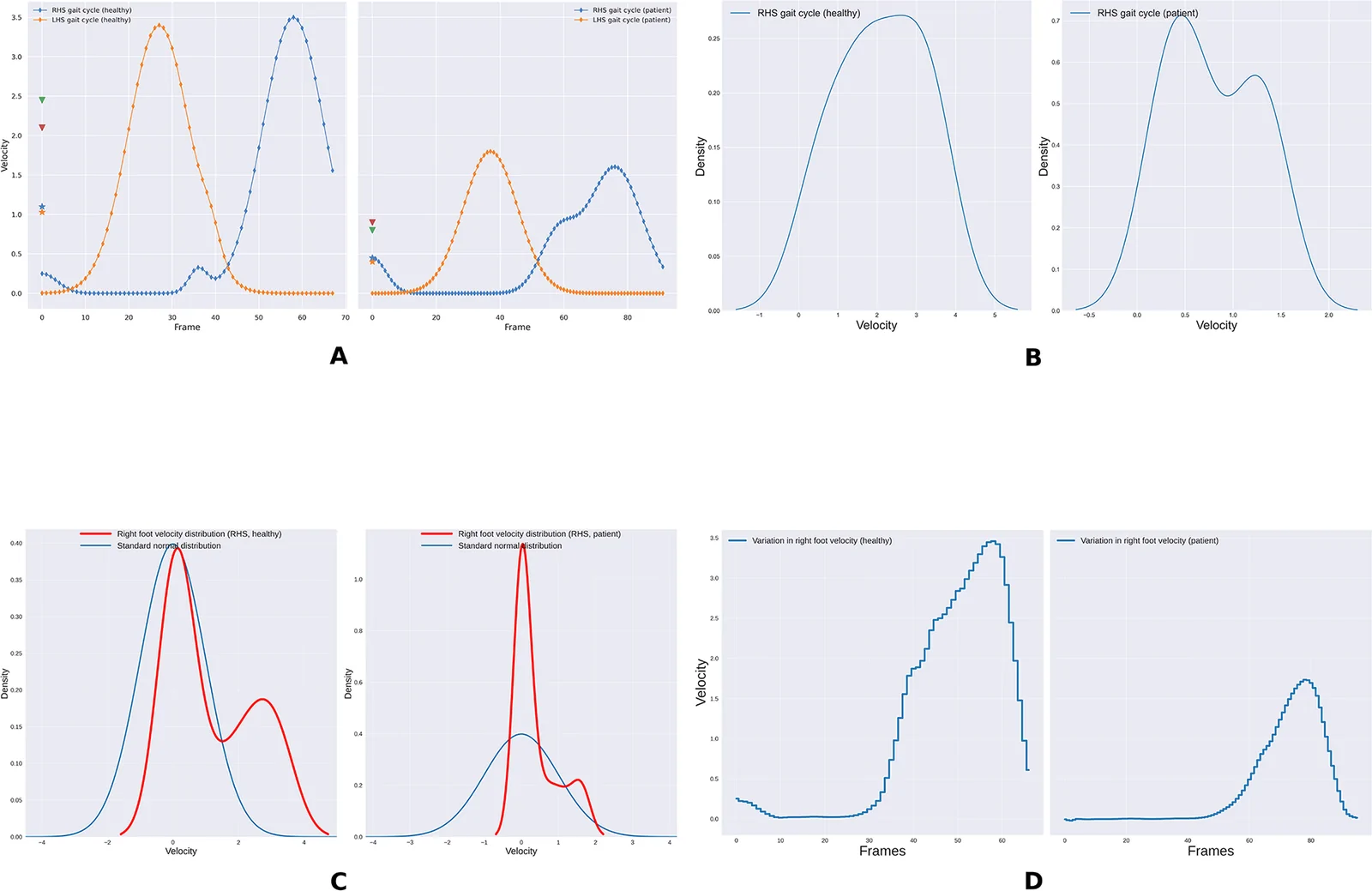
Gait pattern visualizations based on velocity. (A) Comparison of right foot velocity across gait cycles and health conditions. The symbol ∇ indicates the first and third quartiles of the corresponding pattern, and the symbol ⋆ indicates the median and mean. (B) Kernel density estimate of the combined left and right velocities during the right-heel-strike gait cycle for both healthy individuals and patients with stroke. (C) Comparison between the right foot velocity distribution and the standard normal distribution. (D) Variations in right foot velocity during the right-heel-strike gait cycle for both healthy individuals and patients with stroke.

The statistics module summarized gait trajectories using commonly used statistical measures, including range, maximum, mean, median, SD, and the 25th and 75th percentiles (IQR), as previously described [36]. These statistical descriptors were used to provide cycle-level summaries that were less dependent on the starting point of the gait cycle. As illustrated in Figure 2, gait cycles initiated from different events, such as right heel strike and left heel strike, may have different temporal representations despite reflecting comparable movement patterns.

To further characterize gait trajectory patterns, skewness, kurtosis, and total variation were incorporated into the statistics module. Skewness was used to summarize distributional asymmetry, kurtosis to describe the peakedness of the distribution, and total variation to depict joints’ ranges of motion across the gait cycle. These additional descriptors were intended to capture complementary characteristics of poststroke gait patterns beyond the standard statistical measures. The captured IMU data were categorized into joint position and joint angle modalities, and separate models were trained for each modality. The output probabilities from both modalities were then combined to generate the final prediction.

#### Contrastive Learning

In the subsequent phase of the SA Head module, the global prototype for label *k* is refined by incorporating the prototypes of true-positive (TP) samples in *D* through an exponential moving average. Let $D_{TP}^{k}$ be the set of TP samples for label *k* in a batch with size $n_{TP}^{k}$. The exponential moving average operation can be defined as follows:


(1) $$P_{k}=(1-\alpha )\cdot \frac{1}{n_{TP}^{k}}\sum_{i\in D_{TP}^{k}}F_{i}+\alpha \cdot P_{k}$$

where *F_i_* is the prototype of the *i*th sample, and *P_k_* is the global prototype for label *k*. *P_k_* will converge to a consistent representation after training. For the false-negative (FN) and false-positive (FP) samples of label *k*, only a local cluster at the center is computed. Similarly, for the 2 types of samples, the cluster at the center is calculated:


(2) $$\mu_{FP}^{k}=\frac{1}{n_{FP}^{k}}\sum_{i\in D_{FP}^{k}}F_{i},\;\mu_{FN}^{k}=\frac{1}{n_{FN}^{k}}\sum_{i\in D_{FN}^{k}}F_{i}$$

The FN and FP samples, which are often referred to as ambiguous samples, are calibrated by increasing the distance between the TP prototype and the FP samples, while decreasing the distance between the TP prototype and the FN samples. Given a confident TP sample *F_i_*, the compensation term for the FN samples is as follows:


(3) $$\phi_{i}=\left\{ \begin{array}{ll} 1-\mathrm{dis}\left(F_{i},\mu_{FN}^{k}\right) & \text{if }i\in I_{TP}^{k}\wedge n_{FN}^{k}§gt;0\\ 0 & \text{otherwise} \end{array}\right.$$

The penalty term for the FP samples is as follows:


(4) $$\beta_{i}=\left\{ \begin{array}{ll} 1+\mathrm{dis}\left(F_{i},\mu_{FP}^{k}\right), & \text{if }i\in I_{TP}^{k}\wedge n_{FP}^{k}§gt;0\\ 0 & \text{otherwise} \end{array}\right.$$

The cosine similarity function is used to calculate the distance between the TP prototype and the FP samples. The cosine distance is maximized to ensure a shorter distance between the center of the FN cluster and the confident TP samples, thus minimizing the compensation term. Moreover, the penalty term should be minimized. Finally, the contrastive learning loss for the confident sample *F_i_* is defined as follows:


(5) $$\begin{array}{l} CL(F_{i})=-\log\;\left(\frac{e^{\mathrm{dis}(F_{i},P_{k})/\tau -(1-p_{ik})\beta_{i}}}{e^{\mathrm{dis}(F_{i},P_{k})/\tau -(1-p_{ik})\beta_{i}}+\sum_{j\neq k}e^{\mathrm{dis}(F_{i},P_{k})/\tau }}\right)-\\ \log\;\left(\frac{e^{\mathrm{dis}(F_{i},P_{k})/\tau -(1-p_{ik})\phi_{i}}}{e^{\mathrm{dis}(F_{i},P_{k})/\tau -(1-p_{ik})\phi_{i}}+\sum_{j\neq k}e^{\mathrm{dis}(F_{i},P_{k})/\tau }}\right) \end{array}$$

Here, *p*_*ik*_ is the probability of the sample *F_i_* for label *k*, and *τ* is the temperature. The final training objective is to simultaneously minimize the cross-entropy and contrastive learning loss.

#### Model Interpretation Analysis

To quantitatively evaluate the faithfulness of the STAT-Attention module, we conducted an attention-guided occlusion analysis using the trained participant-level 5-fold cross-validation models. For each WGS item, attention weights were aggregated into a 2×4 grid representing 2 modalities, joint position and joint angle, and 4 lower-limb joint groups, including upper leg, lower leg, foot, and toe. For each test participant, these modality-joint cells were ranked according to their attention weights. Single-cell occlusion was then performed by masking the input features corresponding to the selected cell. Four occlusion conditions were examined: the highest-attention cell, the second-highest-attention cell, the lowest-attention cell, and the average across the remaining cells. Faithfulness was evaluated by calculating the decrease in true-class probability after occlusion. A larger decrease indicated that the occluded feature group was more important to the model’s prediction.

### Statistical Analysis

Model performance was evaluated at the participant level. Accuracy was defined as the proportion of predicted WGS item scores that exactly matched the expert reference scores. Because WGS item scores are ordinal, mean absolute error (MAE), linear weighted κ, and quadratic weighted κ were additionally calculated to account for the magnitude of disagreement between predicted and reference scores. Item-level performance was reported for WGS items 2 to 14, and overall performance was calculated by pooling all participant-level item predictions across these items.

To provide a robust estimate of model generalizability, performance metrics were summarized across the participant-level 5-fold cross-validation folds as the mean (SD). The distribution of expert reference scores for each WGS item was also reported to characterize potential class imbalance. To contextualize model performance against expert-level agreement, interrater reliability between the 2 experienced physiotherapists was assessed using their independent ratings. ICCs were calculated using a 2-way random-effects model with absolute agreement and single measures (ICC [2,1]) for each WGS item, the total WGS score, and the summed score of WGS items 2 to 14.

### Data Availability

The raw clinical gait data associated with this study are not publicly available because they contain participant-level movement recordings and are subject to ethical and institutional data-sharing restrictions. Deidentified processed data may be made available from the corresponding author upon reasonable request and subject to institutional approval.

## Results

This study included 51 patients with stroke and 18 healthy individuals. The mean age was 60.3 (SD 10.2) years in healthy individuals and 62.7 (SD 9.5) years in patients with stroke. The poststroke duration was 9.0 (SD 7.2) years. In this sample, 21 patients with stroke and 9 healthy individuals were men. The median FMA-LE score was 24 (IQR 21-29), and the median WGS score was 22 (IQR 18-26; Table 1).

**Figure 3.**
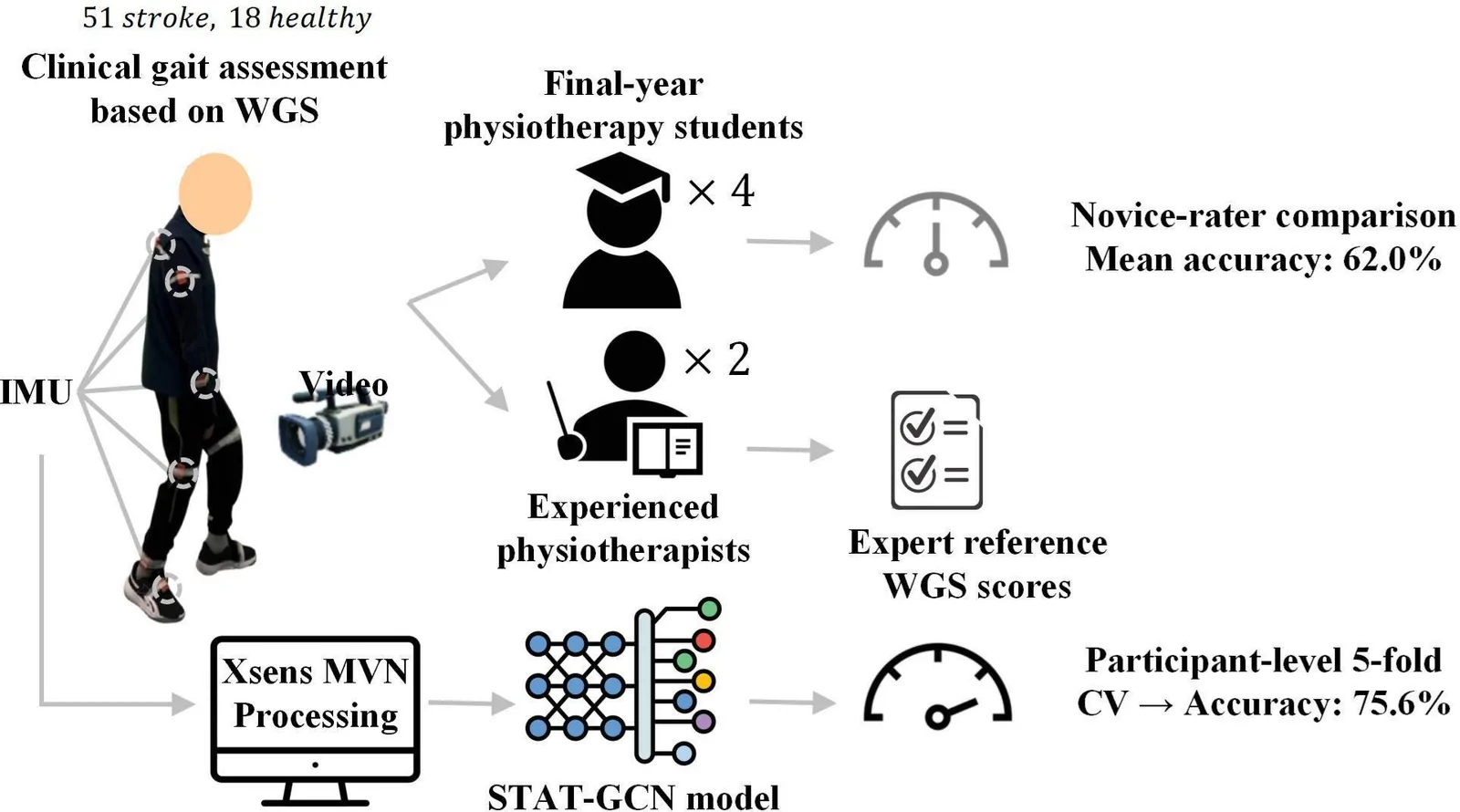
Overview of the gait assessment workflow and performance comparison. CV: cross-validation; IMU: inertial measurement units; MVN: inertial motion capture system; STAT-GCN: statistical graph convolutional network; WGS: Wisconsin Gait Scale.

Figure 3 summarizes the experimental workflow and results. Using instrumented gait data as input, we achieved higher accuracy in WGS ratings compared to 4 final-year physiotherapy students. As presented in Table 2, the STAT-Net model was descriptively compared with 4 final-year physiotherapy students across the participant-level 5-fold cross-validation test sets. The STAT-Net model achieved a mean exact accuracy of 75.64% (SD 6.58%), whereas the 4 student evaluators achieved mean accuracies of 67.09% (SD 4.03%), 65.90% (SD 6.50%), 56.19% (SD 7.03%), and 58.72% (SD 4.22%), respectively. These findings suggest that the model showed higher exact agreement with the expert reference scores than the student evaluators in this novice-rater comparison.

Table 3 presents the item-level prediction performance of STAT-Net among participants with stroke. Across WGS items 2 to 14, the model achieved an overall exact accuracy of 75.42%, with an MAE of 0.290 score levels. The overall linear and quadratic weighted κ values were 0.625 and 0.660, respectively, indicating moderate ordinal agreement with the expert reference scores.

**Table 1.** Characteristics of the participants (N=69).

Characteristics	Stroke	Healthy
Gender (male:female)	24:27	8:10
Side of hemiplegia (right:left)	23:28	N/A^a^
Type of stroke (ischemic:hemorrhagic)	34:17	N/A
Age (y), mean (SD)	62.74 (9.5)	60.3 (10.2)
Height (m), mean (SD)	1.63 (0.09)	1.66 (0.09)
Weight (kg), mean (SD)	65.77 (12.12)	64.10 (12.98)
BMI (kg/m^2^), mean (SD)	23.76 (4.03)	23.11 (3.20)
Poststroke duration (y), mean (SD)	9.0 (7.2)	N/A
FMA-LE^b^, median (IQR)	24 (21-29)	N/A
WGS^c^, median (IQR)	22 (18-26)	N/A

a Not applicable.

b FMA-LE: Fugl-Meyer Assessment for the Lower Extremity.

c WGS: Wisconsin Gait Scale.

**Table 2.** Comparison of Wisconsin Gait Scale (WGS) item classification accuracy (%) between the STAT-Net model and 4 final-year physiotherapy students across the participant-level 5-fold cross-validation test sets.

Method	Fold 0	Fold 1	Fold 2	Fold 3	Fold 4	5-fold mean (SD)
STAT-Net	73.08	82.05	67.52	70.94	84.62	75.64 (6.58)
Evaluator A	65.38	66.67	60.68	71.79	70.94	67.09 (4.03)
Evaluator B	65.38	70.94	55.56	63.25	74.36	65.90 (6.50)
Evaluator C	48.46	55.56	48.72	65.81	62.39	56.19 (7.03)
Evaluator D	57.69	57.26	52.14	62.39	64.10	58.72 (4.22)

**Table 3.** Item-level prediction performance of the model among patients with stroke (n=51).

WGS^a^ item	Accuracy (%)	MAE^b^	Linear weighted κ	Quadratic weighted κ
Item 2	76.47	0.235	0.673	0.749
Item 3	80.39	0.275	0.486	0.357
Item 4	68.63	0.392	0.493	0.472
Item 5	76.47	0.294	0.534	0.570
Item 6	78.43	0.235	0.638	0.726
Item 7	72.55	0.333	0.529	0.527
Item 8	64.71	0.392	0.377	0.387
Item 9	88.24	0.118	0.805	0.860
Item 10	86.27	0.137	0.717	0.740
Item 11	66.67	0.392	0.617	0.710
Item 12	70.59	0.314	0.443	0.513
Item 13	70.59	0.431	0.419	0.320
Item 14	80.39	0.216	0.759	0.812
Overall (N=663)	75.42	0.290	0.625	0.660

a WGS: Wisconsin Gait Scale.

b MAE: mean absolute error.

Item-level performance varied across WGS items. Higher agreement was observed for items 9, 10, and 14, whereas items 8 and 13 showed relatively lower weighted agreement. The distribution of expert reference scores for WGS items 2 to 14 is presented in Table S1 of Multimedia Appendix 2. The score distribution was imbalanced, with lower score categories more frequently represented and severe score categories underrepresented. Score 4 was observed only for item 11 and was absent from the remaining items. The 2 experienced physiotherapists demonstrated high interrater reliability, with ICC (2,1) values of 0.981 for the total WGS score and 0.975 for the summed score of WGS items 2 to 14. Item-level ICC (2,1) values ranged from 0.829 to 0.944, as shown in Table S2 of Multimedia Appendix 2.

The training and testing loss curves for representative WGS items are shown in Figure S1 in Multimedia Appendix 2. The training loss generally decreased across epochs, whereas the testing loss remained relatively stable without clear late-stage divergence, providing supporting evidence for stable model convergence.

**Figure 4.**
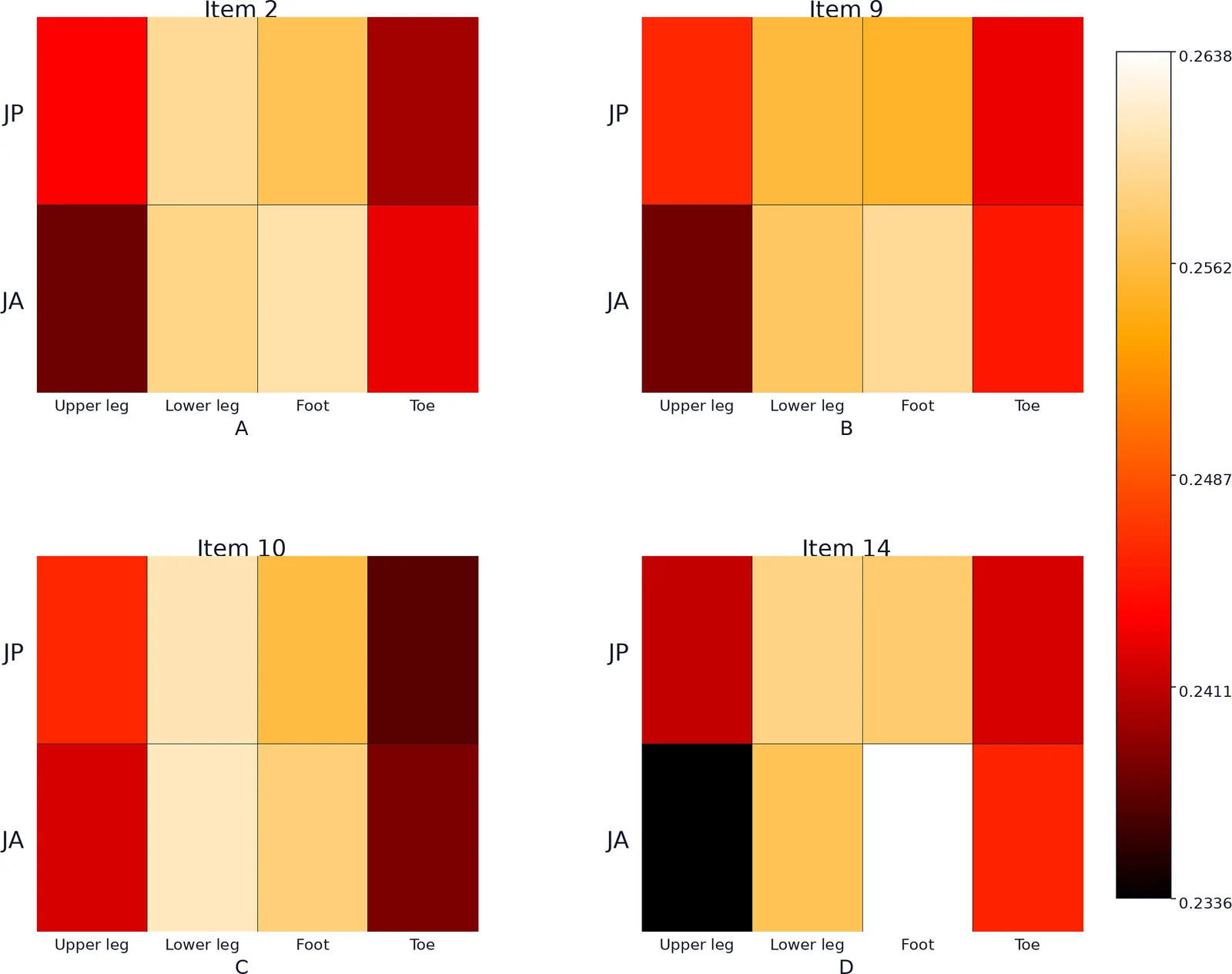
STAT attention heatmaps for selected Wisconsin Gait Scale items. (A-D) Post-Softmax attention heatmaps for WGS Items 2, 9, 10, and 14, respectively. The heatmaps visualize how the model distributes attention across lower-limb joints in the joint-angle (JA) and joint-position (JP) modalities, where warmer colors indicate higher normalized attention weights and suggest the regions that contribute more strongly to the corresponding item prediction.

To provide an exploratory interpretation of the learned attention mechanism, we performed qualitative attention-weighted visualizations and a post hoc feature occlusion analysis. As illustrated in Figure 4, the attention heatmaps show the normalized weights assigned to different lower-limb feature groups across representative WGS items. In each subplot, the rows represent joint-related feature types, and the columns represent lower-limb regions, including the upper leg, lower leg, foot, and toe. Darker pixels indicate lower-attention weights, whereas lighter pixels indicate higher-attention weights. The attention maps show item-specific weighting patterns across lower-limb regions. For example, the model assigned relatively higher weights to foot-related features for item 14, which is consistent with the clinical relevance of foot position during initial contact. These visualizations suggest that the model assigns different levels of priority to lower-limb features across WGS items.

**Figure 5.**
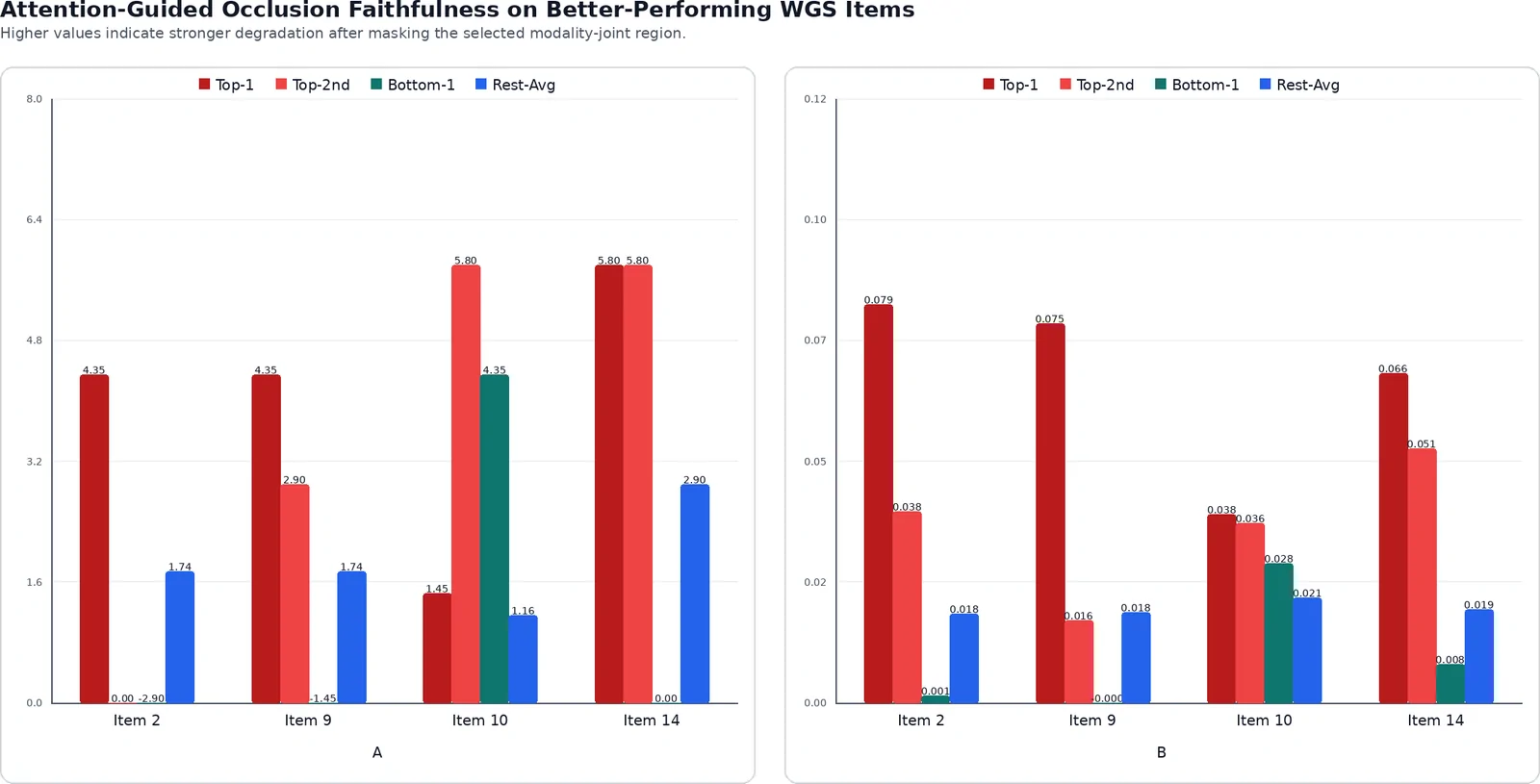
Attention-guided occlusion analysis for selected Wisconsin Gait Scale (WGS) items. (A) Participant-level accuracy drop (%); drop relative to baseline 5-fold test accuracy. (B) True-class confidence drop (decrease in participant-level true-class probability after occlusion).

To further evaluate whether the learned attention weights reflected functionally important input features, an attention-guided occlusion analysis was performed for representative well-performing WGS items. As shown in Table 4 and Figure 5, occluding the highest-attention feature group generally produced larger decreases in true-class probability than occluding the lowest-attention feature group or the remaining feature groups. This pattern was most evident for items 2, 9, and 14. For item 2, top-1 occlusion produced the largest decrease in true-class probability (Δp_true=0.0792), compared with top-2nd (0.0381), bottom-1 (0.0015), and rest-avg (0.0178). Similar patterns were observed for item 9 and item 14. Item 10 showed a less distinct pattern, suggesting that attention faithfulness may vary across WGS items.

**Table 4.** Single-cell attention-guided occlusion faithfulness on selected Wisconsin Gait Scale (WGS) items^a^.

Item	Top-1 Δp_true	Top-2nd Δp_true	Bottom-1 Δp_true	Rest-avg Δp_true
Item 2	0.0792	0.0381	0.0015	0.0178
Item 9	0.0754	0.0164	−0.0001	0.0180
Item 10	0.0375	0.0358	0.0277	0.0210
Item 14	0.0655	0.0505	0.0077	0.0186

a Δp_true indicates the decrease in the predicted probability of the true class after occlusion. Top-1 and Top-2nd refer to the modality-joint cells with the highest and second-highest-attention weights, respectively. Bottom-1 refers to the cell with the lowest-attention weight. Rest-avg represents the average occlusion effect across the remaining cells. Larger positive values indicate a greater reduction in true-class probability after masking and therefore suggest a greater functional contribution of the corresponding feature group to the model prediction.

## Discussion

### Principal Findings

In this study, we developed and evaluated STAT-GCN, a deep learning framework for automated WGS item prediction using IMU-derived kinematic data. Using a stratified, participant-level 5-fold cross-validation protocol, all gait cycles from the same participant were kept within the same fold to reduce the risk of intraparticipant information leakage. The findings suggest that kinematic data-driven models may provide preliminary support for more standardized WGS item scoring; however, they should be interpreted cautiously, given the limited sample size and absence of external validation. Previous work on digital WGS assessment has also suggested that technology-assisted approaches may help reduce subjectivity in observational gait evaluation [37].

The comparison with 4 final-year physiotherapy students was interpreted as a descriptive novice-rater comparison. In this dataset, the model showed higher agreement with the expert reference scores than did the novice raters. However, this comparison should be interpreted cautiously, as the students represent a novice-rater benchmark rather than an expert clinical standard. The high interrater reliability between the 2 experienced physiotherapists indicates that expert-level WGS assessment remains more consistent than that of the current automated model. Therefore, the findings should not be interpreted as evidence that STAT-GCN has achieved expert-level clinical performance. Instead, the model may provide preliminary support for more standardized WGS item scoring, particularly when expert raters are unavailable or when repeated assessments are required.

Item-level performance varied across WGS items, suggesting that some gait components were more difficult for the model to predict than others. Relatively low agreement was observed for items 8 and 13, both of which involve rotational components. Item 8 assesses external rotation during the initial swing phase of the affected leg, whereas item 13 assesses pelvic rotation during terminal swing. These transverse-plane movements may be challenging because they are difficult to estimate visually and may also be sensitive to sensor placement and movement variability. In addition, item 11, which assesses knee flexion from toe-off to mid-swing by comparing the affected and unaffected limbs, may require more explicit bilateral and phase-specific information than the current model input provides. Future model development may benefit from incorporating clinically informed features, such as inter-limb ratios, phase-specific joint trajectories, or additional transverse-plane kinematic descriptors.

The distribution of expert reference scores also indicated class imbalance across WGS items. Lower score categories were more frequently represented, whereas severe score categories were underrepresented. This imbalance may have affected both model learning and performance interpretation, because the model had fewer examples from which to learn severe gait deviations. Similar concerns have been reported in other gait-related machine learning studies, where class imbalance and limited representation of severe cases were found to influence model performance and stability, highlighting the importance of balanced datasets and appropriate augmentation strategies [38]. It may also explain why exact accuracy alone could provide an incomplete picture of model performance. For this reason, ordinal metrics such as MAE and weighted κ were included to better account for the magnitude of disagreement between predicted and expert reference scores. Future studies should include larger and more clinically diverse samples and may consider class-balanced training strategies, cost-sensitive learning, or carefully validated data augmentation methods.

The attention-guided occlusion analysis provided partial quantitative support for the faithfulness of the STAT-Attention module. In several representative WGS items, masking high-attention modality-joint regions produced larger reductions in true-class probability than masking low-attention regions, suggesting that the model relied more heavily on features assigned higher attention weights. However, this pattern was item-dependent and should not be interpreted as complete evidence of clinical explainability. In addition, aggregating attention weights into a 2×4 modality-joint grid may obscure more detailed joint-specific and time-specific information. Therefore, the attention maps should be considered supportive indicators of feature relevance rather than definitive explanations of the model’s decision-making process. Future studies should compare this approach with established explainability methods and further examine whether the highlighted features correspond to specific clinically defined WGS gait deviations.

Although automated WGS item prediction may help reduce subjectivity and improve the standardization of repeated gait assessments, the present study did not directly examine whether model-predicted WGS scores improve rehabilitation decision-making, treatment planning, or clinical outcomes. Therefore, the translational value of the proposed framework remains preliminary. Future studies should investigate whether automated item-level WGS predictions can be linked to individualized exercise prescription, longitudinal treatment response, or clinically meaningful functional recovery.

### Limitations

This study has several limitations. First, although participant-level cross-validation and training-only data augmentation were used to reduce the risk of overly optimistic performance estimates, the sample size remained limited for deep learning model development, and no external validation dataset was available. The limited sample size and a relatively low representation of participants with severe gait impairment may have affected model stability, particularly for higher WGS score categories, and may limit generalizability to broader populations of individuals with stroke and more diverse gait impairments. Independent validation across different clinical sites, devices, and participant populations is required before clinical implementation. Second, the model relied primarily on kinematic data. Additional modalities, such as plantar pressure, force-plate measurements, or video-based movement features, may improve the prediction of items related to weight shifting, limb comparison, and transverse-plane rotation. However, the use of additional equipment may reduce clinical feasibility, and future work should balance model performance with practical usability.

### Conclusions

STAT-GCN demonstrated reasonable preliminary performance for automated WGS item prediction in individuals with stroke using IMU-derived kinematic data. By combining a graph-based representation of lower-limb kinematics with participant-level validation, this study provides initial evidence that data-driven models may support more standardized observational gait assessment. However, further studies using larger and more clinically diverse stroke cohorts, additional data modalities, and independent external validation are needed to determine the clinical utility and generalizability of this approach.

## Supplementary material

10.2196/94031 Multimedia Appendix 1 Wisconsin Gait Scale full items.

10.2196/94031 Multimedia Appendix 2 Wisconsin Gait Scale score distributions and interrater reliability.

10.2196/94031 Checklist 1 STROBE checklist.

## Abbreviations

FMA-LE: Fugl-Meyer Assessment of the Lower Extremity
FN: false-negative
FP: false-positive
GCN: graph convolutional network
ICC: intraclass correlation coefficient
IMU: inertial measurement unit
MAE: mean absolute error
ST-GCN: spatio-temporal graph convolutional network
STAT-GCN: statistical graph convolutional network
STROBE: Strengthening the Reporting of Observational Studies in Epidemiology
TP: true-positive
WGS: Wisconsin Gait Scale

## References

[1] Wafa HA, Wolfe CDA, Emmett E, Roth GA, Johnson CO, Wang Y (2020). Burden of stroke in Europe: thirty-year projections of incidence, prevalence, deaths, and disability-adjusted life years. Stroke.

[2] Pu L, Wang L, Zhang R, Zhao T, Jiang Y, Han L (2023). Projected global trends in ischemic stroke incidence, deaths and disability-adjusted life years from 2020 to 2030. Stroke.

[3] Heshmatollah A, Darweesh SKL, Dommershuijsen LJ, Koudstaal PJ, Ikram MA, Ikram MK (2020). Quantitative gait impairments in patients with stroke or transient ischemic attack: a population-based approach. Stroke.

[4] Pan JW, Sidarta A, Wu TL (2024). Unraveling stroke gait deviations with movement analytics, more than meets the eye: a case control study. Front Neurosci.

[5] Lattouf NA, Tomb R, Assi A, Maynard L, Mesure S (2021). Eccentric training effects for patients with post-stroke hemiparesis on strength and speed gait: a randomized controlled trial. NeuroRehabilitation.

[6] Wang Y, Mukaino M, Ohtsuka K (2020). Gait characteristics of post-stroke hemiparetic patients with different walking speeds. Int J Rehabil Res.

[7] Kwong PW, Ng SS, Liu TW, Chung RC, Ng GY (2016). Effect of leg selection on the Berg Balance Scale scores of hemiparetic stroke survivors: a cross-sectional study. Arch Phys Med Rehabil.

[8] Kwong PWH, Ng SSM, Chung RCK, Ng GYF (2017). A structural equation model of the relationship between muscle strength, balance performance, walking endurance and community integration in stroke survivors. PLoS One.

[9] Li J, Kwong PWH, Li H, Tham T, Fong KNK, Wang L (2025). Validating gait profile score for post-stroke gait: correlation with observational scales and clinical outcomes. Physiother Pract Res.

[10] Kerr A, Rowe P, Clark A (2020). Biomechanical correlates for recovering walking speed following a stroke. The potential of tibia to vertical angle as a therapy target. Gait Posture.

[11] Guzik A, Drużbicki M, Wolan-Nieroda A, Przysada G, Kwolek A (2019). The Wisconsin Gait Scale—the minimal clinically important difference. Gait Posture.

[12] Yaliman A, Kesiktas N, Ozkaya M, Eskiyurt N, Erkan O, Yilmaz E (2014). Evaluation of intrarater and interrater reliability of the Wisconsin Gait Scale with using the video taped stroke patients in a Turkish sample. NeuroRehabilitation.

[13] Pandis N (2014). Introduction to observational studies: part 2. Am J Orthod Dentofacial Orthop.

[14] Li J, Kwong PWH, Lua EK, Chan MYL, Choo A, Donnelly CJW (2023). Development of a convolutional neural network (CNN) based assessment exercise recommendation system for individuals with chronic stroke: a feasibility study. Top Stroke Rehabil.

[15] Bivard A, Churilov L, Parsons M (2020). Artificial intelligence for decision support in acute stroke—current roles and potential. Nat Rev Neurol.

[16] Hu K, Wang Z, Martens KAE (2021). Graph fusion network-based multimodal learning for freezing of gait detection. IEEE Trans Neural Netw Learning Syst.

[17] Sabo A, Mehdizadeh S, Iaboni A, Taati B (2022). Estimating parkinsonism severity in natural gait videos of older adults with dementia. IEEE J Biomed Health Inform.

[18] Yan S, Xiong Y, Lin D (2018). Spatial temporal graph convolutional networks for skeleton-based action recognition. Proc AAAI Conf Artif Intell.

[19] Zhang X, He L, Chen K, Luo Y, Zhou J, Wang F (2018). Multi-view graph convolutional network and its applications on neuroimage analysis for Parkinson’s disease. AMIA Annu Symp Proc.

[20] Li M, Chen S, Chen X, Zhang Y, Wang Y, Tian Q Actional-structural graph convolutional networks for skeleton-based action recognition.

[21] Zhang X, Xu C, Tao D Context aware graph convolution for skeleton-based action recognition.

[22] Huang L, Huang Y, Ouyang W, Wang L (2020). Part-level graph convolutional network for skeleton-based action recognition. Proc AAAI Conf Artif Intell.

[23] Shahroudy A, Liu J, Ng TT, Wang G NTU RGB+D: a large scale dataset for 3D human activity analysis.

[24] Scheper MC, van Velzen M, van Meeteren NLU (2024). Towards responsible use of artificial intelligence in daily practice: what do physiotherapists need to know, consider and do?. J Physiother.

[25] Yu BXB, Liu Y, Zhang X, Chen G, Chan KCC EGCN: an ensemble-based learning framework for exploring effective skeleton-based rehabilitation exercise assessment.

[26] Li J, Kwong PW, Lin W, Fong KN, Wu W, Sidarta A (2025). Assessment of ambulation functions through kinematic analysis in individuals with stroke: a systematic review. Eur J Phys Rehabil Med.

[27] Myn U, Link M, Awinda M (2015). Xsens MVN user manual. https://www.xsens.com/hubfs/Downloads/usermanual/MVN_User_Manual.pdf.

[28] Rodriquez AA, Black PO, Kile KA (1996). Gait training efficacy using a home-based practice model in chronic hemiplegia. Arch Phys Med Rehabil.

[29] Fugl-Meyer AR, Jääskö L, Leyman I, Olsson S, Steglind S (1975). The post-stroke hemiplegic patient. 1. A method for evaluation of physical performance. Scand J Rehabil Med.

[30] Hernández ED, Forero SM, Galeano CP, Barbosa NE, Sunnerhagen KS, Alt Murphy M (2021). Intra- and inter-rater reliability of Fugl-Meyer Assessment of Lower Extremity early after stroke. Braz J Phys Ther.

[31] Sullivan KJ, Tilson JK, Cen SY (2011). Fugl-Meyer assessment of sensorimotor function after stroke: standardized training procedure for clinical practice and clinical trials. Stroke.

[32] Twitchell TE (1951). The restoration of motor function following hemiplegia in man. Brain.

[33] (2023). Sensor placement in Xsens Awinda system. Xsens.

[34] Um TT, Pfister FMJ, Pichler D Data augmentation of wearable sensor data for Parkinson’s disease monitoring using convolutional neural networks.

[35] Zhou H, Liu Q, Wang Y Learning discriminative representations for skeleton based action recognition.

[36] Dong W, Yuan T, Yang K, Li C, Zhang S Autoencoder regularized network for driving style representation learning.

[37] Guzik A, Wolan-Nieroda A, Drużbicki M (2022). Assessment of agreement between a new application to compute the Wisconsin gait score and 3-dimensional gait analysis, and reliability of the application in stroke patients. Front Hum Neurosci.

[38] Trabassi D, Castiglia SF, Bini F (2024). Optimizing rare disease gait classification through data balancing and generative AI: insights from hereditary cerebellar ataxia. Sensors (Basel).

