# Supplementary material for: Deep Learning–Based Automated Clinical Gait Assessment From Kinematic Data in People With Stroke: Development and Validation Study
Source: JMIR Rehabil Assist Technol. 2026 Aug 19;13:e94031. doi: 10.2196/94031 (PMC13489110; doi:10.2196/94031)
Supplement: Supplementary file 2 [file rehab-v13-e94031-s002.docx]

**Appendix 2**

Supplementary Table S1. Distribution of expert reference WGS item scores among stroke participants.

|  | Score 1 (n) | Score 1 (%) | Score 2 (n) | Score 2 (%) | Score 3 (n) | Score 3 (%) | Score 4 (n) | Score 4 (%) |
| --- | --- | --- | --- | --- | --- | --- | --- | --- |
| Item 2 | 12 | 23.5 | 25 | 49 | 14 | 27.5 | 0 | 0 |
| Item 3 | 33 | 64.7 | 14 | 27.5 | 4 | 7.8 | 0 | 0 |
| Item 4 | 17 | 33.3 | 21 | 41.2 | 13 | 25.5 | 0 | 0 |
| Item 5 | 35 | 68.6 | 11 | 21.6 | 5 | 9.8 | 0 | 0 |
| Item 6 | 33 | 64.7 | 12 | 23.5 | 6 | 11.8 | 0 | 0 |
| Item 7 | 26 | 51 | 18 | 35.3 | 7 | 13.7 | 0 | 0 |
| Item 8 | 22 | 43.1 | 23 | 45.1 | 6 | 11.8 | 0 | 0 |
| Item 9 | 32 | 62.7 | 15 | 29.4 | 4 | 7.8 | 0 | 0 |
| Item 10 | 15 | 29.4 | 32 | 62.7 | 4 | 7.8 | 0 | 0 |
| Item 11 | 11 | 21.6 | 21 | 41.2 | 13 | 25.5 | 6 | 11.8 |
| Item 12 | 31 | 60.8 | 16 | 31.4 | 4 | 7.8 | 0 | 0 |
| Item 13 | 24 | 47.1 | 19 | 37.3 | 8 | 15.7 | 0 | 0 |
| Item 14 | 18 | 35.3 | 15 | 29.4 | 18 | 35.3 | 0 | 0 |

Supplementary Table S2. Inter-rater reliability between two experienced physiotherapists for WGS item scores and total scores.

| **Measure** | **Item 2** | **Item 3** | **Item 4** | **Item 5** | **Item 6** | **Item 7** | **Item 8** | **Item 9** | **Item 10** | **Item 11** | **Item 12** | **Item 13** | **Item 14** | **Total WGS score** | **Sum of Items 2–14** |
| --- | --- | --- | --- | --- | --- | --- | --- | --- | --- | --- | --- | --- | --- | --- | --- |
| ICC(2,1) | 0.895 | 0.883 | 0.854 | 0.843 | 0.931 | 0.829 | 0.931 | 0.880 | 0.836 | 0.917 | 0.838 | 0.944 | 0.889 | 0.981 | 0.975 |

Supplementary Figure S1: Training versus Testing Loss Curves.
