# Supplementary material for: Deep Learning–Based Automated Clinical Gait Assessment From Kinematic Data in People With Stroke: Development and Validation Study
Source: JMIR Rehabil Assist Technol. 2026 Aug 19;13:e94031. doi: 10.2196/94031 (PMC13489110; doi:10.2196/94031)
Supplement: Supplementary file 3 [file rehab-v13-e94031-s001.docx]

**Appendix 1** Wisconsin Gait Scale full items
